# A Robust Approach to Quantify Nocifensive Blink Reflex Responsiveness

**DOI:** 10.1111/ejn.70421

**Published:** 2026-02-17

**Authors:** R. J. Bufacchi, R. Somervail, K. Shao, M. Kilintari, G. Novembre, G. D. Iannetti

**Affiliations:** ^1^ International Center for Primate Brain Research (ICPBR) Chinese Academy of Sciences Shanghai Branch Shanghai China; ^2^ Neuroscience and Behaviour Laboratory Italian Institute of Technology (IIT) Center for Life and Nano Science Rome Italy; ^3^ Epigenetics & Neurobiology Unit European Molecular Biology Laboratory Rome Monterotondo Italy; ^4^ Translational and Computational Neuroscience Unit Manchester Metropolitan University Manchester UK; ^5^ Department of Neuroscience, Physiology and Pharmacology University College London (UCL) London UK; ^6^ Department of Psychology, Faculty of Science and Technology Bournemouth University Bournemouth UK; ^7^ Neuroscience of Perception and Action Laboratory Italian Institute of Technology (IIT) Center for Life and Nano Science Rome Italy

**Keywords:** electromyography (EMG), hand‐blink reflex (HBR), nervous system, nocifensive behaviour, responder classificationresponse variability

## Abstract

The modulation of the hand‐blink reflex (HBR), a prototypical nocifensive response, is increasingly used to investigate defensive behaviour, related to the notion of peripersonal space. However, HBR responsiveness is highly variable across participants. This variability has led researchers to use several seemingly arbitrary criteria to determine whether a subject should be included as a participant in a study. But are these criteria justified? Can better and more rigorous criteria be formulated? Does the traditional division into responders and non‐responders reflect a practical decision to exclude participants with very low signal‐to‐noise ratio, or does it reflect two distinct biological categories? Here, we addressed these issues by systematically varying a set of parameters, which together form an objective and quantifiable criterion of HBR responsiveness. We describe classification criteria for HBR responsiveness that were both reliable and consistent with previous studies. We also found no evidence for a clear‐cut biological distinction between HBR responders and non‐responders. We recommend to (1) no longer preliminarily screen subjects, simply collecting data on all subjects, and (2), after collecting the data, only include subjects identified as blinkers using the following criteria: The mean of the rectified HBR must exceed 2.5 SD of the baseline EMG in 40% or more of trials in the hand‐near condition. We formulate rigorous inclusion criteria for HBR studies, which can be adapted for use on other neurophysiological responses in health and disease.

## Graphical abstract

The hand‐blink reflex (HBR) is used to investigate defensive behaviour and peripersonal space, but its application is limited because it is highly variable across participants, and we lack robust criteria to determine whether a participant should be included in the study. Here, we describe reliable classification criteria for HBR responsiveness: The integral of the rectified HBR must exceed 2.5 SD of the baseline EMG in 40% or more of trials in the hand‐near condition. We recommend to no longer screen subjects for HBR responsiveness but instead collect data from all participants, and subsequently only statistically analyse data from participants identified as HBR responders.

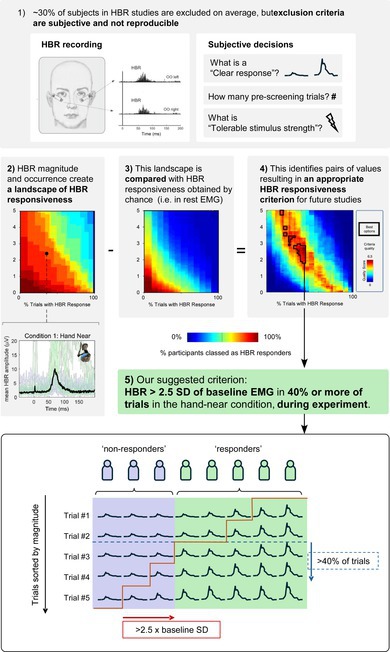

**Abbreviations**
EEG: electroencephalography
EMG: electromyography
HBR: hand‐blink reflex
SNR: signal‐to‐noise ratio

## 1 Introduction

The hand‐blink reflex (HBR) is evoked by electrical stimulation of the median nerve at the wrist, and is recorded as an electromyographic (EMG) response from the orbicularis oculi muscles, bilaterally (Valls‐Solé et al. 1997). HBR magnitude depends on the position of the stimulated hand in head‐centred coordinates, mapping out a field of action relevance surrounding the face (Sambo, Liang, et al. 2012; Sambo and Iannetti 2013; Bufacchi et al. 2016). This method has led to several novel insights regarding the way the nervous system assigns behavioural relevance to proximal environmental events. For example, HBR fields are affected by physical barriers (Sambo, Forster, et al. 2012), anxiety (Sambo and Iannetti 2013), gravitational cues (Bufacchi and Iannetti 2016), motion of threats (Wallwork et al. 2016; Bisio et al. 2017; Bufacchi 2017; Somervail et al. 2019), interpersonal interactions (Fossataro, Gindri, et al. 2016; Fossataro, Sambo, et al. 2016), chronic pain conditions (Bufacchi et al. 2017), blindness (Wallwork et al. 2017), protective postures (Biggio et al. 2019), visuomotor feedback and intention (Fossataro et al. 2018, 2020), and emotional context and history (Fossataro et al. 2023; Mercante et al. 2024).

However, an important issue when using the HBR remains unaddressed: In standard laboratory conditions, not all subjects respond to somatosensory stimulation with an HBR strong enough to be empirically useful. That is, in some participants, the HBR magnitude is so small that they are considered to be ‘non‐responders’ (Valls‐Solé et al. 1997; Miwa et al. 1998). HBR studies report 20%–50% of participants to be non‐responders (Miwa et al. 1995, 1998; Valls‐Solé et al. 1997; Álvarez‐Blanco et al. 2009; Sambo, Forster, et al. 2012; Sambo, Liang, et al. 2012; Sambo and Iannetti 2013; Bufacchi et al. 2016; Bufacchi and Iannetti 2016; Fossataro, Gindri, et al. 2016; Fossataro, Sambo, et al. 2016; Wallwork et al. 2016; Bisio et al. 2017). This issue is particularly important if experimentally varied factors contribute to whether an individual is classified as HBR responsive.

The variability in population responsiveness raises several questions, which carry both pragmatic and physiological interest. How effective and useful are the current classification criteria? Can we formulate a better, more rigorous criterion than those currently used? Importantly, when we ask whether an individual should be classified as a responder, we are asking whether HBR magnitude is *large enough* to be helpful in investigating experimental questions. However, this does also raise another question of biological importance: Is an individual classified as a responder based on an arbitrary cut‐off, or is there a real boundary observable in the population? In other words, is the classification purely pragmatic or indicative of some important physiological distinction?

The criterion to classify an individual as a responder should be based on the expected characteristics of a consistent response: It should be large enough to be reliably measured and occur in enough trials to make any statistical tests meaningful. The current procedure to decide whether an individual is an HBR responder involves increasing the stimulus current until ‘a clear response is observed in three consecutive trials, or the participant refused a further increase of stimulus intensity’ (Valls‐Solé et al. 1997; Sambo, Liang, et al. 2012) (note that here ‘stimulus intensity’ was used to mean stimulus current rather than perceived intensity). Participants showing a ‘reproducible HBR’ are considered responders (Valls‐Solé et al. 1997; Sambo, Liang, et al. 2012).

**FIGURE 1:**
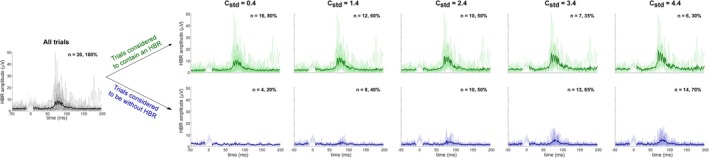
Effect of C_std_ criterion values on labelling trials as ‘containing an HBR’ or ‘without HBR’. Data from a representative participant. The left plot shows all 20 single HBR trials with their corresponding across‐trial average. The plots to the right show progressively more stringent C_std_ criteria and their effect on the number of single trials labelled as ‘containing’ or ‘without’ an HBR. Thick lines show averages across trials of the two categories. Time around zero is greyed out because of the presence of the stimulation artefact.

There are three clear issues with this procedure. First, the number of consecutive trials (3) is arbitrary and quite low. Second, it does not define the inter‐stimulus interval, which is important given that the HBR habituates rapidly. Third, and most important, what an experimenter should consider a ‘clear HBR’ is undefined: How large must the response be for it to be ‘clear’? (Figure 1).

Here, we attempted to address these questions by suggesting an objective and quantifiable method of determining HBR responsiveness that is both reliable and consistent with previous studies.^1^ Specifically, researchers should collect HBR data on all subjects and after the data collection only include subjects for whom the mean of the rectified HBR in the hand‐near condition exceeds 2.5 SD of the baseline EMG in ≥ 40% of trials. We found these specific criteria and demonstrated their validity by analysing empirical data from four HBR experiments. Two of these experiments were performed on all recruited subjects, regardless of preliminary screening for an individual's HBR responsiveness. This allowed us to obtain a full sampling of HBR responsiveness across the general population. The other two experiments were performed only on HBR responders identified through currently used criteria.

## 2 Materials and Method

In this manuscript, we report an analysis of data from four separately collected experiments. Experiments 1 and 2 were designed to address two questions at the same time. The first question (for both experiments) was the issue of HBR responsiveness, which is detailed in the current manuscript. The second question differed between Experiments 1 and 2. In Experiment 1, currently unpublished, the question concerned the relationship between electroencephalographic (EEG) activity and HBR responsiveness. In Experiment 2, the question concerned the relationship between physical effort and HBR magnitude (Bufacchi et al. 2019). Data for Experiments 3 and 4 were collected before this project was conceptualised. Experiments 2–4 have already been published (Bufacchi and Iannetti 2016; Bufacchi et al. 2019); hence, the present work represents a reanalysis of the data from those experiments. We collected data from a total of 100 participants across the four experiments.

To ensure consistency across analyses of data from the different experiments, we only extracted those experimental conditions that were common to at least two experiments.

All experimental procedures were approved by the local ethical committee, and all participants gave their permission to use their data in both the original and subsequent studies, as specified in the information sheet and consent form.

### 2.1 Participants

We collected data from 39, 19, 21 and 21 participants for Experiments 1–4, respectively (17 women, 22.0 ± 3.1 years; 14 women, 23.6 ± 4.3 years; 12 women, 26.3 ± 5.8 years; 12 women, 26.2 ± 5.7 years). In Experiments 3 and 4, we only collected data from participants who were previously identified as HBR responders using the old procedure, whereas in Experiments 1 and 2, we collected data from all participants who were willing to participate, without screening them for HBR responsiveness.

### 2.2 Stimulation and Recording

Stimulation and recording procedures are detailed elsewhere (Sambo, Liang, et al. 2012; Bufacchi et al. 2016). Briefly, intense electrical stimuli were delivered transcutaneously to the median nerve at the wrist. EMG activity was recorded from the orbicularis oculi muscle bilaterally using surface electrodes. In Experiments 1–4, participants received 80, 48, 50 and 50 stimuli (inter‐stimulus interval ~30 s) across 4, 2, 2 and 2 blocks, respectively. Stimulus duration was 200 μs.

In all experiments, before each session of testing, we established the maximum tolerable intensity of stimulation for each participant. This was achieved by incrementally increasing the current of the electrical stimulus until the subject either refused a further increase or reported that the sensation had become painful. If the sensation was painful, the intensity was turned down until the subject no longer experienced pain. At this point, the current of this stimulus was noted, and all stimuli in the rest of the experiment were delivered at this stimulus intensity. Therefore, none of the participants reported painful sensations, even at high stimulus intensities.

### 2.3 Experimental Protocol

To ensure consistency across analyses of data from the different experiments, we only extracted the two experimental conditions that were common to at least two experiments.

**FIGURE 2:**
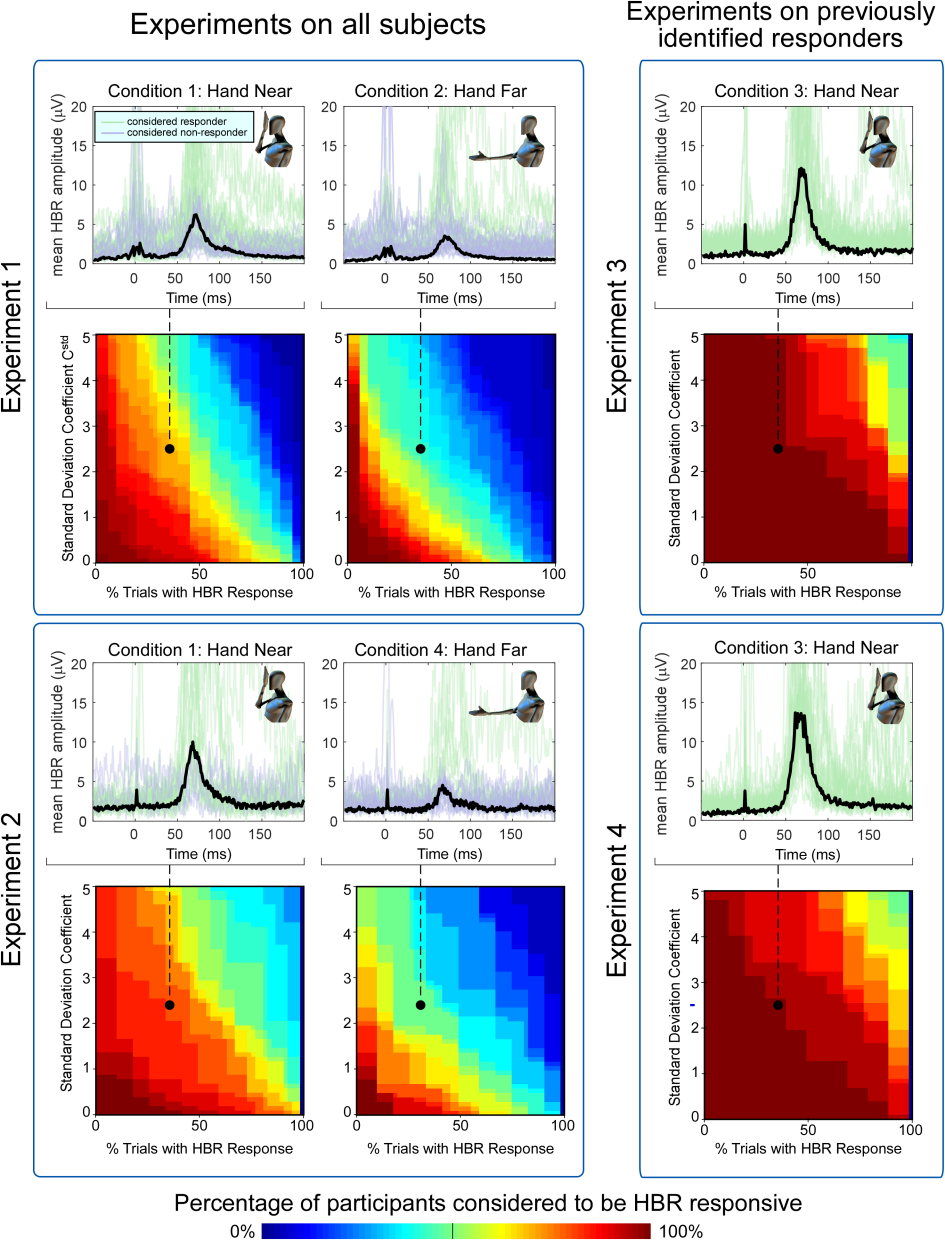
Effect of pairs of criterion values on estimated HBR responsiveness. The combination of parameters $c^{\mathrm{std}}$ and $c^{\mathrm{per}}$ forms an objective and quantifiable criterion of HBR responsiveness. $c^{\mathrm{std}}$ represents the minimum number of standard deviations that the HBR should be larger than the prestimulus EMG to be considered a stimulus‐evoked response. $c^{\mathrm{per}}$ represents the percentage of trials in which a blink occurs for a subject to be classified as responder. In each box, the top row of figures shows average HBR responses for each subject (blue and green lines), and the grand average across subjects (black line). Blue lines indicate participants labelled as ‘non‐responders’ based on the criteria highlighted with a black dot in the colour maps on the row below (C_STD_ = 2.5, C_per_ = 40%), whereas green lines indicate participants labelled as ‘responders’. The colour maps show the percentage of subjects judged to be responders as a function of the combination of used criteria. Experiments 1 and 2 (left blue boxes) were performed on all subjects, regardless of preliminary screening for responsiveness. This allowed us to obtain a full sampling of the HBR responsiveness across the general population. Experiments 3 and 4 (right blue boxes) were performed only on participants previously identified as HBR responders through already existing criteria. In all Experiments, making either of the parameters $c^{\mathrm{std}}$ or $c^{\mathrm{per}}$ less stringent resulted in a continuous increase of participants classified as HBR responders, between 0% and 100%.

Specifically, in these two experimental conditions, electrical stimuli were delivered to the median nerve of the right hand. The right hand was held either in the ‘far’ position (~60 cm from the eyes, with the arm extended and resting on a table in front of the participant) or in the ‘near’ position (~4 cm from the eyes) (Figure 2). Experiments 3 and 4 did not include the ‘far’ condition; hence, only the ‘near’ condition was extracted from these experiments.

Experiments 1 and 2 consisted of four blocks each, two of which were analysed in the present work. Experiment 2 has been described elsewhere (Bufacchi et al. 2019), as have Experiments 3 and 4 (Bufacchi and Iannetti 2016). For more details about the questions behind each experiment and the experimental conditions not included in this study, see Table 1.

**TABLE 1:** Details of the experiments.

Exp no.	Published (Y/N)	Experimental question (original reason for conducting experiment)	Excluded conditions
1	N	What are the EEG correlates of the increase in HBR magnitude when the hand is near the face? Identify objective criteria for including participants in HBR studies	Two control conditions were excluded from the present analysis. In these conditions, the stimulated hand remained far from the face, whereas the non‐stimulated hand was alternated between the far and the near positions. This provided a control for EEG effects that were not related to stimulation
2	Y (Bufacchi et al. 2019)	Does muscular effort contribute to HBR magnitude? Identify objective criteria for including participants in HBR studies	Two conditions were excluded from the present analysis. In these conditions, a 1‐kg weight was placed on the stimulated hand, whereas the stimulated hand remained far from the face, either resting on a table (Condition 1) or lifted 1 cm above the table (Condition 2). This provided an estimate of the effect of pressure on the hand (Condition 1) and muscular effort (Condition 2) on HBR magnitude
3	Y (Bufacchi and Iannetti 2016)	How is the HBR magnitude modulated by stimulus position along the horizontal body axis?	Four conditions were excluded from the present analysis. In these conditions, the hand was placed at the same antero‐posterior and vertical position from the face as the ‘near’ condition, but along different horizontal locations. This provided estimates of the effect of horizontal hand displacement on HBR magnitude
4	Y (Bufacchi and Iannetti 2016)	How is the HBR magnitude modulated by stimulus position along the vertical body axis?	Four conditions were excluded from the present analysis. In these conditions, the hand was placed at the same antero‐posterior and horizontal position from the face as the ‘near’ condition, but along different vertical locations. This provided estimates of the effect of vertical hand displacement on HBR magnitude

### 2.4 Data Analyses and Statistics

#### 2.4.1 Preprocessing

EMG signals from each participant were high‐pass filtered (55 Hz) and full‐wave rectified. HBR magnitude was calculated as the mean EMG amplitude of each single‐trial response between 50 and 90 ms post‐stimulus, separately for each eye. This procedure is described in further detail in (Sambo, Liang, et al. 2012).

#### 2.4.2 Criteria to Quantify HBR Responders

In brief, we used the magnitude of EMG activity in a pre‐stimulus time‐window (−50 to −10 ms) as reference, and the magnitude of EMG activity in a post‐stimulus window (50–90 ms) as potential HBR response. We then compared these values to identify whether or not a response in the post‐stimulus time window was present in each trial (using the $c^{\mathrm{std}}$ criterion; see below and Figures 1 and 2). We subsequently used the percentage of trials with an identified HBR, within experimental condition and subject, to determine whether or not a subject should be considered a ‘responder’ (using the $c^{\mathrm{per}}$ criterion; see below and Figure 2).

More specifically, for each experiment *e*, each subject *s* and each experimental condition *c*, in the pre‐stimulus time window, we computed the mean $M_{e,s,c}^{\mathrm{pre}}$ and standard deviation ${\mathrm{SD}}_{e,s,c}^{\mathrm{pre}}$ of the rectified EMG magnitude across time and trials. For each experiment, subject, experimental condition and trial *t*, we also computed the mean EMG during the post‐stimulus response time window across time $M_{e,s,c,t}^{\text{post}}$. These measures allowed us to calculate the number of ‘observable HBRs’ $B_{e,s,c}$ for each experiment, subject and experimental condition as

$$B_{e,s,c}=\sum_{t}\left\{\begin{array}{c} 1{\mathrm{ifM}}_{e,s,c,t}^{\text{post}}>M_{e,s,c}^{\mathrm{pre}}+c^{\mathrm{std}}\cdot S_{e,s,c}^{\mathrm{pre}}\\ 0{\mathrm{ifM}}_{e,s,c,t}^{\text{post}}\leq M_{e,s,c}^{\mathrm{pre}}+c^{\mathrm{std}}\cdot S_{e,s,c}^{\mathrm{pre}} \end{array}\right.$$

where $c^{\mathrm{std}}$ is the criterion that determines how many standard deviations larger than baseline activity the activity during the response window must be for a trial t to be considered an HBR. From the number of observable HBRs, we then computed the number of participants classified as an HBR responder $N_{e,c}$ as

$$N_{e,c}=\sum_{s}\left\{\begin{array}{c} 1\text{if}\frac{B_{e,s,c}}{T_{e,s,c}}>\frac{c^{\mathrm{per}}}{100}\\ 0\text{if}\frac{B_{e,s,c}}{T_{e,s,c}}\leq \frac{c^{\mathrm{per}}}{100} \end{array}\right.$$

where $T_{e,s,c}$ is the total number of trials for a given experiment, subject and condition and $c^{\mathrm{per}}$ is the criterion that determines *the percentage of trials with an HBR that must be observed to classify a subject as HBR responder* in a specific condition.

In this way, we used two criteria ($c^{\mathrm{std}}$ and $c^{\mathrm{per}}$) to determine *whether a subject is an HBR responder*. For each experiment and condition, we varied these criterion parameters from 0 to 5 ($c^{\mathrm{std}}$) and from 0% to 100% ($c^{\mathrm{per}}$) and observed the percentage of participants that were identified as HBR responders.

#### 2.4.3 Permutation Testing

To compare the resulting percentages of responders to the percentage of apparent HBR responders one would expect by chance if there were no true responders, we ran permutation tests for each experiment and condition. These random percentages of HBR responders were obtained by performing the same procedure described above, but using EMG data sampled randomly from all timepoints outside the prestimulus and response time windows defined above (note that we used the same number of timepoints as in the response time window). By repeating this procedure 1000 times on these randomly sampled time windows, we built a null distribution of the number of participants classified as HBR responders given non‐response data, for each combination of criterion parameters. This allowed us to calculate, for each cut‐off criterion, (1) how many *more* participants were classified as responders than if we used non‐response data and (2) the probability of observing that percentage or a higher percentage by chance (i.e., a *p*‐value).

#### 2.4.4 Testing for Unimodality of HBR Responsiveness

We also formally tested for bimodality in the population response magnitude. If present, bimodality would indicate a clear‐cut distinction between groups of individuals with different response properties (i.e., responders and non‐responders). We first conducted, in each subject, unpaired *t*‐tests between the mean EMG during the response time window $M_{e,s,c,t}^{\text{post}}$ and the mean EMG during the pre‐stimulus time window $M_{e,s,c,t}^{\mathrm{pre}}$. We then constructed two separate histograms describing the frequency distribution of the magnitudes of the *t*‐statistics across subjects. One histogram contained subjects that represented the general population (Experiments 1 and 2). The population comprising the other histogram represented participants labelled as HBR responders using traditional criteria (Experiments 3 and 4), for which there should be no bimodal distribution, and hence, Experiments 3 and 4 served as a sanity check. Finally, we checked the resulting histograms for unimodality of the distribution, using Hartigan's dip test (Hartigan and Hartigan 1985). If there were distinct responder and non‐responder populations, we expected the null hypothesis of unimodality to be rejected.

#### 2.4.5 Anxiety Correlation

To test whether a participant's psychological trait anxiety predicted their HBR responsiveness, we conducted Pearson's correlation tests between scores of trait anxiety (as assessed by the trait subscale of the State–Trait Anxiety Inventory; Spielberger et al. 1970), and the percentage of trials classified as an HBR, for each value of $c^{\mathrm{std}}$.

## 3 Results

### 3.1 Quantification of HBR Responders

In Experiments 1 and 2 (conducted on all subjects regardless of preliminary screening for HBR responsiveness), making either $c^{\mathrm{std}}$ or $c^{\mathrm{per}}$ less stringent resulted in a smoothly increasing percentage of participants classified as HBR responders (Figure 2). This result provides a first piece of evidence that there is no binary separation between responders and non‐responders. That is, the distinction of responders versus non‐responders should be thought of as a pragmatic rather than a biological separation: saying that somebody is a responder should be taken as meaning that their HBR magnitude is *large enough* to be helpful in investigating the given experimental questions.

Importantly, the percentage of responders identified under a specific pair of criterion values depended on the position of the hand that was stimulated to elicit the HBR. This indicates that it is imperative to define which hand position is used to test for HBR responsiveness, because a participant might be classified as a responder when their hand is in one position, but not when it is in another. In Experiments 3 and 4, where the participants had already been screened for HBR responsiveness, we also found that the percentage of participants classified as responders ranged continuously from 0% to 100%, depending on how strict the criteria used were. In other words, if criteria are stringent, even subjects identified as HBR responders using the traditional method of Valls‐Solé et al. (1997) become non‐responders. This is a further suggestion that there is no clear separation between responders and non‐responders and that these labels should be taken as reflecting a decision based on practical utility, rather than biological classification.

### 3.2 Finding New HBR Responsiveness Criteria

**FIGURE 3:**
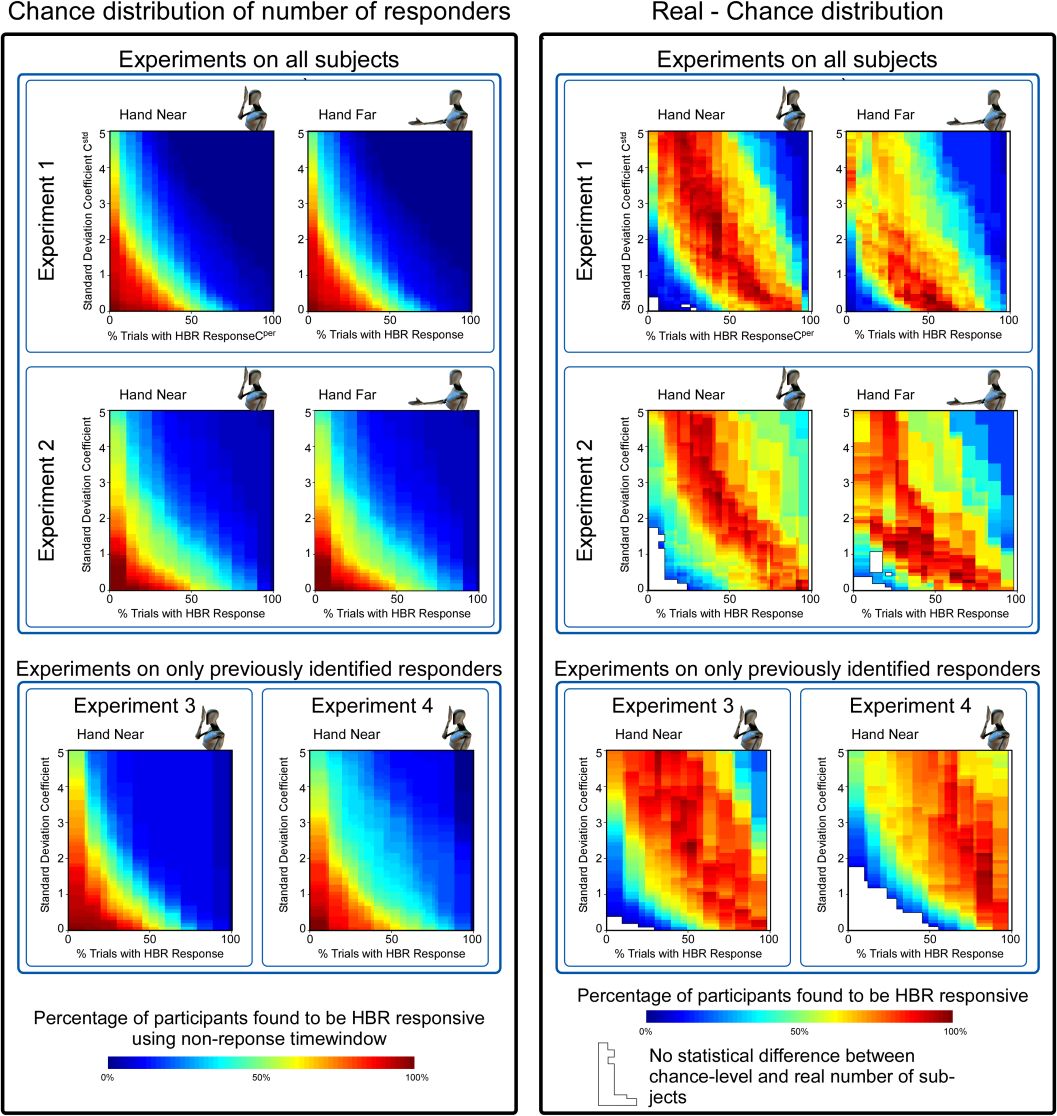
Comparison of population responsiveness to chance. Left panel: By randomly sampling EMG activity from timepoints outside the HBR response window we built a null‐distribution of the number of participants classified as HBR responders if there were no response being sampled (i.e., a chance‐level distribution). Right panel: Comparison between the number of participants classified as HBR responders under a set of specific criteria, to the corresponding chance distribution. This approach allowed us to calculate (1) how many *more* participants were classified as responders in comparison with chance (colour maps in right panels) and (2) the probability (expressed as *p*‐value) of observing that percentage by chance. White patches indicate pairs of criteria for which there is no difference between real responder numbers and those arising by chance.

Permutation testing showed that for almost any pair of response criteria (i.e., $c^{\mathrm{std}}$ > ~ 0.3, and $c^{\mathrm{per}}$ between ~10% and ~95%), the percentage of participants classified as responders was larger than the percentage of participants that would be classified as responders by chance (Figure 3). There was no single combination of criteria that clearly yielded an optimal percentage of responders higher than chance (Figure 3, right panel). Nonetheless, when pairs of either very strict or very relaxed criteria were used, there was almost no difference between real and chance‐level classifications, indicating that extreme criteria should not be used.

**FIGURE 4:**
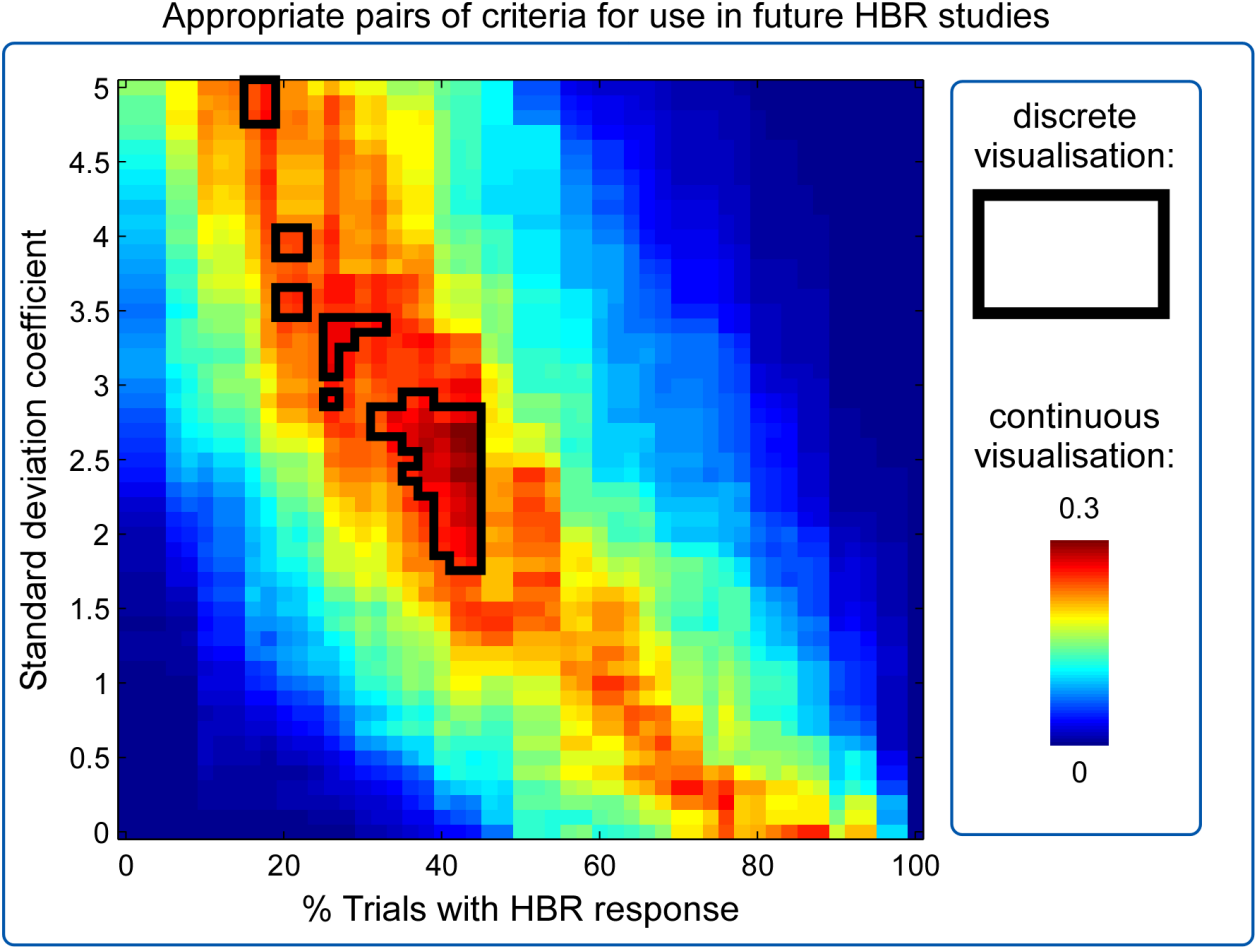
Pairs of values resulting in an appropriate responsiveness criterion for the hand‐near condition. Regions outlined in black show pairs of criteria in which (1) data from Experiments 1 and 2 showed a percentage of responders > 50% higher than chance and (2) data from Experiments 3 and 4 showed a percentage of responders > 95%. Colour coding indicates, for each value of $c^{\mathrm{std}}$ and $c^{\mathrm{per}}$, the multiplication of the percentage of responders higher than chance in Experiments 1 and 2 with the total percentage of responders classified in Experiments 3 and 4. These plots demonstrate that there is a range of $c^{\mathrm{std}}$ and $c^{\mathrm{per}}$ parameters which should be chosen to obtain a responsiveness criterion, which is both consistent with old experiments, as well as able to discriminate a large percentage of the participants more likely to be an HBR responder than by chance.

To further investigate what might be an optimal range of $c^{\mathrm{std}}$ and $c^{\mathrm{per}}$, we pooled data collected from the same ‘near’ condition (i.e., the hand being placed 4 cm from the ipsilateral eye) from all four experiments and then displayed the HBR responsiveness functions in two separate ways. First, we identified the pairs of criteria in which (1) data from Experiments 1 and 2 showed a percentage of responders > 50% higher than chance and (2) data from Experiments 3 and 4 showed a percentage of responders > 95% higher than chance. In this way, the data from Experiments 1 and 2 allowed identifying those pairs of criteria that yieldedg the maximal difference from non‐response data *when recording all subjects*, whereas the data from Experiments 3 and 4 allowed identifying those pairs of criteria that resulted in the selection of the same number of responders *compared with the previously employed criteria* (regions enclosed by black lines in Figure 4). Second, we multiplied, for each value of $c^{\mathrm{std}}$ and $c^{\mathrm{per}}$, the percentage of responders higher than chance in Experiments 1 and 2 by the total percentage of responders classified in Experiments 3 and 4. This similarly allowed us to visualise which parameter values resulted in the most consistent and discriminative criterion values, in a graded fashion (colour maps in Figure 4) rather than in a discrete fashion (regions enclosed by black lines; Figure 4). These two methods of visualisation clearly demonstrate that there is a range of $c^{\mathrm{std}}$ and $c^{\mathrm{per}}$ parameters, which could be chosen to obtain a responsiveness criterion that is both consistent with old experiments, as well as able to identify a larger group of participants than is likely by chance.

### 3.3 Testing for Unimodality of HBR Responsiveness

**FIGURE 5:**
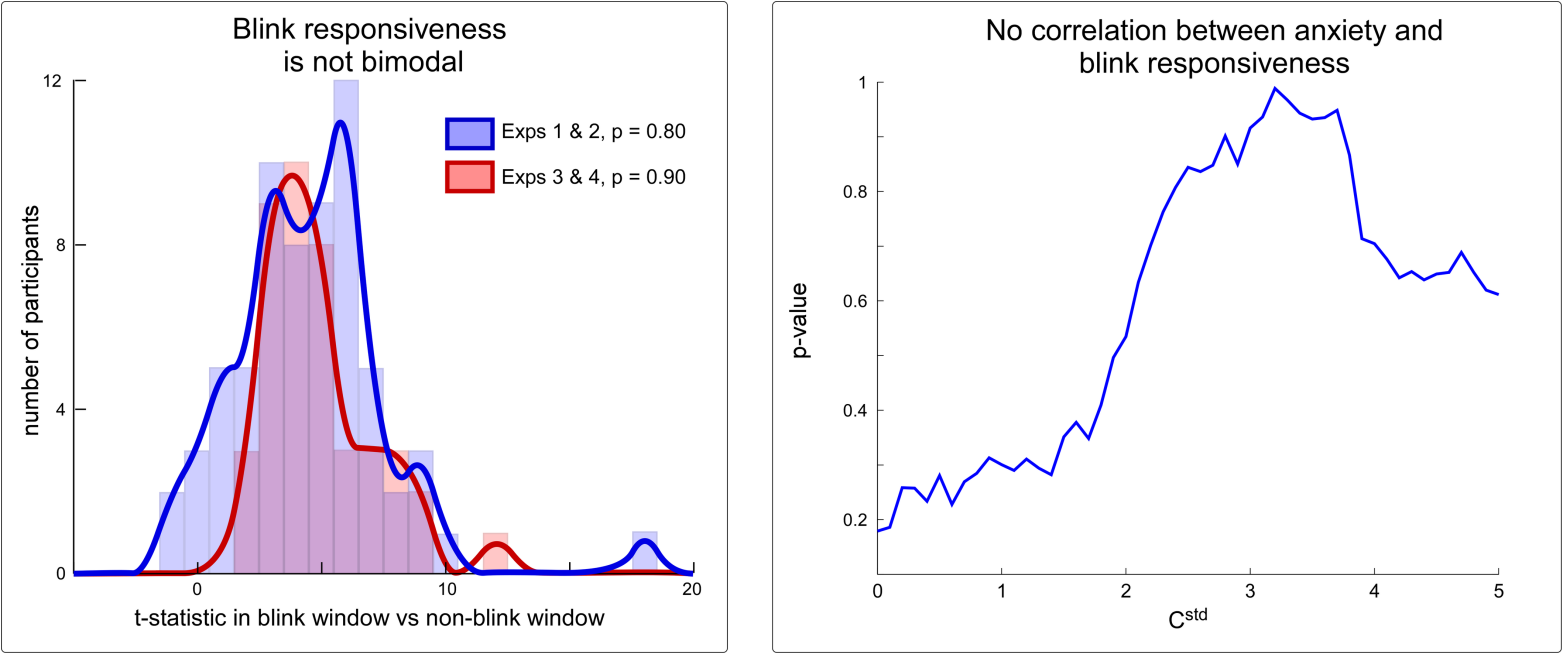
Distribution of HBR responsiveness across the general population. Left panel: Testing for population unimodality in responsiveness. We first conducted unpaired *t*‐tests between the mean EMG during the response time‐window and the mean EMG during the pre‐stimulus time window in each subject. The blue and red histograms describe the frequency distribution of the magnitudes of the *t*‐statistics for Experiments 1 and 2 (blue) and Experiments 3 and 4 (red). Hartigans' dip test for distribution unimodality showed no evidence for two distinct populations of subjects (responders and non‐responders), suggesting instead a continuous spectrum of responsiveness across the population. This makes defining a responsiveness criterion more of a matter of practicality than physiological understanding. Importantly, the distinction of responders versus non‐responders should therefore be considered a pragmatic rather than a biological separation: saying that somebody is a responder should be taken as meaning that their HBR magnitude is *large enough* to be helpful in investigating the given experimental questions. Right panel: Lack of correlation between trait anxiety and percentage of HBR responses. There was no robust evidence for a relationship between trait anxiety and the percentage of trials classified as an HBR for any value of C_std_.

Hartigan's dip test showed no evidence against unimodality in either histogram (*p* = 0.80 and *p* = 0.90; Figure 5). This result provides further evidence that there is no clear‐cut biological distinction between responders and non‐responders.

### 3.4 Anxiety Correlation

Pearson's correlation tests showed that there was no robust evidence for a link between trait anxiety and the percentage of trials classified as an HBR, for any value of $c^{\mathrm{std}}$ (*p* > 0.179 for all tests; Figure 5). This result indicates that trait anxiety cannot be used as a reliable preliminary screening tool to identify HBR responders.

## 4 Discussion

Here we have provided the first unbiased description of HBR responsiveness in the general population by showing how the number of responders varies in relation to the classification criteria used.

### 4.1 Recommended Criteria to Assess HBR Responsiveness

A range of parameter values could be chosen to obtain an HBR responsiveness criterion, which is both consistent with old experiments, as well as able to discriminate a large percentage of the participants above chance level (Figures 2, 3, 4). Given that hand position dictates HBR magnitude, which affects the sensitivity of the resulting criterion, the function describing the percentage of responders also depended on the hand position at which the HBR was elicited. For this reason, to assess HBR responsiveness, we suggest using the hand position that results in the largest HBR magnitude, that is, directly in front of the face (Sambo, Liang, et al. 2012; Bufacchi et al. 2016). For simplicity and the sake of maximising the number of data points, we would advise to use the following combinations of values: $c^{\mathrm{std}}=2.5$ and $c^{\mathrm{per}}=40\%$ (when collecting data with roughly 30 s inter‐stimulus intervals, as is the norm in the field). Note that this combination of values is the most lenient to slight deviations, suggesting that it is the most suitable, and should be used across experiments and research groups to standardise results.

Hence, we advise to no longer preliminarily screen subjects for future HBR experiments, but to just collect data on all subjects, using a stimulus intensity that the participant indicates they will be able to tolerate for the duration of the experiment, and then, after collecting the data, to analyse only those subjects identified as blinkers using the combination of criterion values described here on the HBR collected in the ‘near’ hand position, to improve the signal‐to‐noise ratio (SNR) and maintain consistency with earlier studies. Although this approach may seem more time consuming than excluding participants who are considered non‐responders, in fact, it will probably take the same amount of time as current practices and be more beneficial, given that (1) screening subjects is time consuming, (2) most HBR experiments do not last longer than an hour, (3) this method will result in historically consistent figures of approximately 70% of participants being classified as HBR responders, (4) it allows correlates of HBR responsiveness to be investigated across studies and (5) it reduces subjective bias and promotes transparency.

Finally, the difference in optimal criteria between far and near conditions is easily explained. In the far condition, HBR magnitude is substantially smaller, and even strong HBR responders are unlikely to show many trials above $c^{\mathrm{std}}=2.5$. To achieve robust measures of responsiveness in this condition, a lower $c^{\mathrm{std}}$ allows for more trials to be identified. It follows that a concomitant increase in $c^{\mathrm{per}}$ is needed to maintain high SNR. Given that a lower $c^{\mathrm{per}}$ allows identifying responders with fewer total HBR trials, the ‘near’ condition is more beneficial as a criterion than the far.

### 4.2 HBR Responsiveness Falls on a Continuous Spectrum

We found that in all considered experiments and conditions, the percentage of participants classified as HBR responders ranged continuously from 0% to 100%, depending on the chosen values of the pair of classification criteria $c^{\mathrm{std}}$ (i.e., the number of standard deviations above baseline for a response to be considered an HBR; Figures 1 and 2) and $c^{\mathrm{per}}$ (i.e., the minimal percentage of HBRs observed during an experiment to consider a subject HBR responsive; Figure 2). The fact that such a continuous range was also observed in Experiments 3 and 4, where the participants had already been screened for responsiveness using methods employed in previous studies (Bufacchi and Iannetti 2016), further indicates that there is no clear separation between responders and non‐responders. Importantly, what we mean by this is that these labels should be taken as reflecting the practical utility of their data, rather than as a classification of two populations with distinct physiological traits. This lack of separation was also confirmed by the unimodal distribution of the *t*‐statistics comparing baseline and response EMG (Figure 4). These findings are important when interpreting the results from HBR studies: Given that HBR responsiveness seems to be distributed along a continuous spectrum, the neural effects underlying HBR modulations are likely also present in ‘non‐responders’, albeit difficult to quantify.

### 4.3 Correlates of HBR Responsiveness Across Individuals

Despite HBR responsiveness seeming to be more of a continuous function than a step function, some participants are still clearly more responsive than others. Stimulus current, which is related to current density at the median nerve, undoubtedly plays a role; within participants, we often observe that the higher the stimulus current, the stronger the resulting HBR. Therefore, to maximise SNR, stimulus current is often raised until the participant refuses a further increase (Valls‐Solé et al. 1997). However, stimulus current cannot be the only factor in determining HBR responsivity; some participants display strong and repeatable HBRs with stimulus intensities as low as 5 mA, whereas others still do not show a clear HBR even at 99 mA, the maximal output of a standard electrical stimulator. Nonetheless, almost no reliable correlates of HBR responsiveness have been found in healthy participants so far. Here we again failed to find one: There was no correlation between trait anxiety and the number of participants classified as HBR responsive, for any combination of criterion values. This is particularly surprising given that we have previously found a link between the *shape* of the HBR response field and trait anxiety (Sambo and Iannetti 2013).

However, recent work on a larger dataset has shown that the 'avoidance' and 'care' dimensions in certain questionnaires can predict HBR responsiveness (defined using the existing criteria): Responders showed lower scores in ‘avoidance’ and higher scores in the ‘care’ dimensions (Mercante et al. 2024). Such results promise interesting implications once they are further unpacked. One other feature has been found that seems to predict HBR responsiveness in a clinical population: the presence of facial trigger zones in patients affected by trigeminal neuralgia. Trigeminal neuralgia is a condition in which innocuous trigeminal stimulation triggers paroxysmal facial pain. The trigger zones for this pain can lie on the face or inside the mouth, and in a recent study, only 29% of patients with exclusively intraoral triggers were found to be HBR responsive (2 out of 7). In contrast, 82% of patients with extraoral triggers were found to be HBR responsive (18 out of 22) (Bufacchi et al. 2017). Despite the old and unreliable criteria used for estimating these percentages, this result still suggests that HBR responsiveness depends on the amount of behavioural relevance given to stimuli near the face. Other recent work with patient populations has shown that classifiers trained on HBR responses are able to distinguish people with multiple sclerosis from healthy volunteers (Biggio et al. 2022) and that HBR magnitudes are different for migraine sufferers (Ayas et al. 2019), suggesting that it might be possible and informative to find further predictors of HBR responsiveness.

### 4.4 HBR Responsiveness From a Dynamic Systems Perspective

It can be fruitful to consider the HBR and our method of classification from a dynamic systems perspective. The system can be seen as operating in two regimes: the baseline and the response regime. The baseline regime can be envisaged as a stochastic distribution around low‐amplitude activity. Abrupt sensory stimulation then deviates the system into the response regime with high‐amplitude activity. The separation of responders and non‐responders is difficult because the deviation of trajectories caused by stimulation is not fully separated from the stochastic noise of the baseline regime, at least when using EMG to define the state space. The $c^{\mathrm{std}}$ criterion then defines a hyperplane in the state space and trajectories crossing the space above the hyperplane are counted as an HBR trial. We can then clearly see that the true distributions of trajectories might differ between experimental conditions (e.g., different hand positions might be understood as system parameters generating the trajectories). This perspective emphasises that a reproducible method of judging responsiveness is important: A weak criterion may allow researchers to inadvertently tune or game responsiveness criteria to obtain a desired statistical result. In contrast, by numerically assessing the selection criteria, the current method reveals the ergodic structure in a larger range of the state space of the neural system. This potentially links state trajectories to the effects of experimental conditions (in the case of HBR, the possible hand positions) and allows insight into the underlying mechanism of event generation.

### 4.5 Responsiveness in Other Physiological Responses

Finally, the issue of signal detection is by no means exclusive to HBR research. Motor‐evoked potentials, sharp‐wave ripples and event‐related potentials are some of the many neural events to which a similar approach could be applied. These neural events, either stimulus‐driven or spontaneously occurring, are detected using specific detection criteria that incorporate a variety of features. These commonly include spectral elements derived from Fourier or wavelet transforms (Buzsáki 2019), pattern recognition through template matching (Baillet et al. 2007) and more sophisticated features learned via machine learning and deep neural networks. However, only including the subset of events that meet such predefined criteria often leads to a selection bias in the samples of the state space (Shao et al. 2022), as demonstrated by comparing the response landscape in Experiments 3 and 4 versus Experiments 1 and 2 (Figure 1). In contrast, our method allows for a less‐biased sampling of state space, and a potentially better distinction between experimental variables, suggesting that our method might be applicable to other reflexes or biological responses. That said, there are no strict guarantees that the specific criterion values that we identified for the HBR would transfer validly to other reflexes. We hope that the simple idea we put forward will provide a starting point or a proof of concept for future, more general yet still simple methods.

## Author Contributions

R.J.B. and G.D.I. conceptualised the study. R.J.B., R.S. and M.K. collected the data. R.J.B. analysed the data. All authors interpreted the results. R.J.B. and G.D.I. wrote the paper. All authors edited the paper.

## Funding

This work was supported by the European Research Council Consolidator PAINSTRAT Grant (649020) and Shanghai Municipal Human Resources and Social Security Bureau (E35CN31A21).

## Conflicts of Interest

The authors declare no conflicts of interest.

## Data Availability

Empirical data used in this study are available upon reasonable request.

## Endnotes

^1^
Note that this method is specific to HBR and hence not to be used blindly for studying other defensive reflexes.
